# Supplementary material for: Myricetin Amorphous Solid Dispersions—Antineurodegenerative Potential
Source: Molecules. 2024 Mar 14;29(6):1287. doi: 10.3390/molecules29061287 (PMC10975365; doi:10.3390/molecules29061287)
Supplement: Supplementary file 1 [file molecules-29-01287-s001.zip › molecules-2855809-supplementary.pdf]

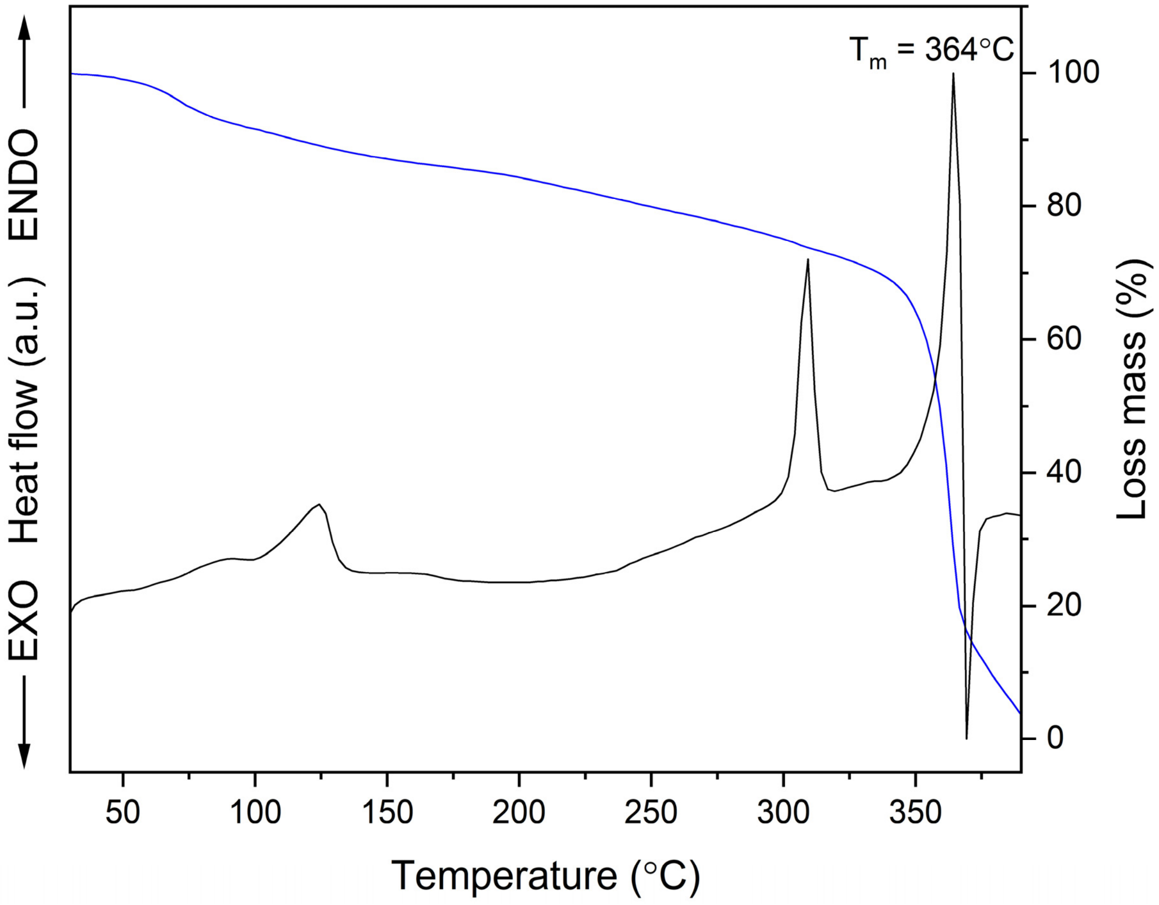

Figure S1. TG analysis of myricetin (blue line) and DSC analysis of myricetin (first heating, black line).

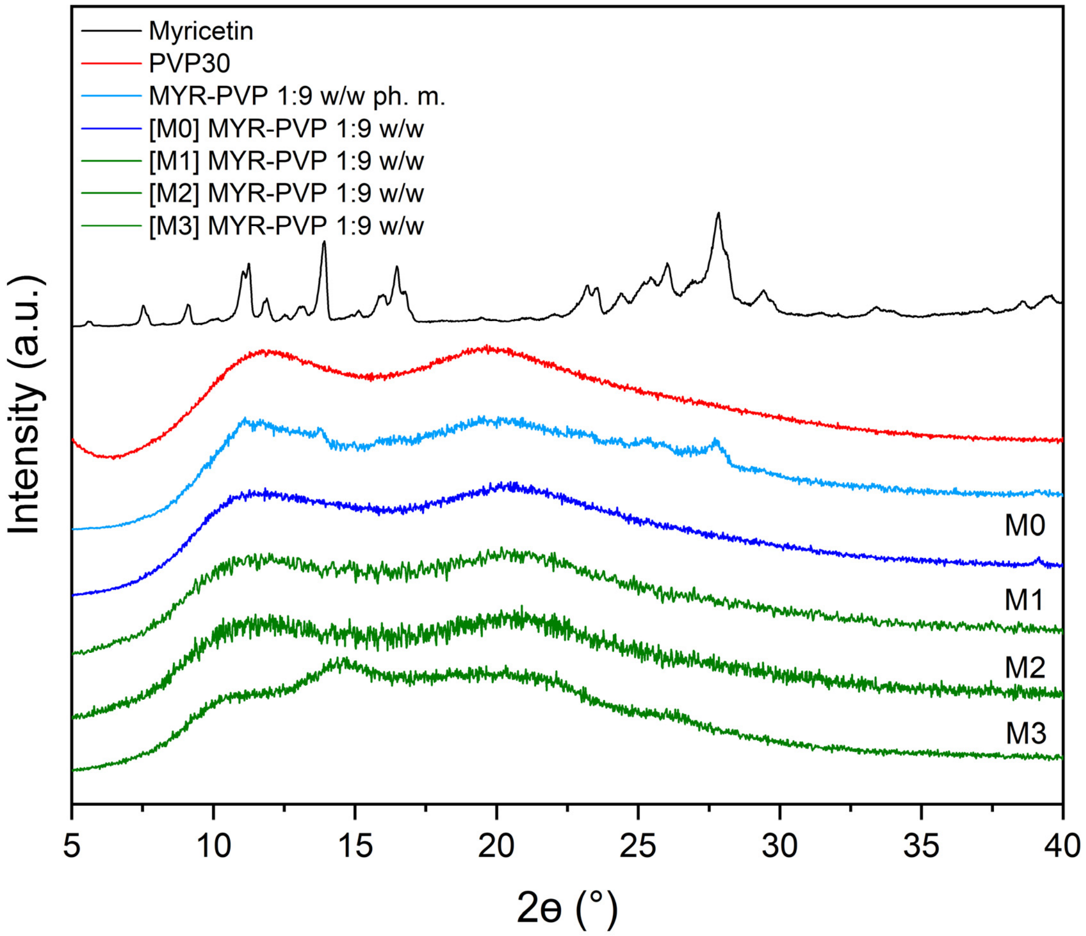

Figure S2. Physical stability studies (XRPD patterns, range 5–40° 2Θ): myricetin (MYR, black line), PVP30 (red line), MYR-PVP30 1:9 w/w physical mixture (light blue line), MYR-PVP30 1:9 w/w ASD (dark blue line, M0), MYR-PVP30 1:9 w/w ASD after after 1, 2 and 3 months (dark green lines, M1–M3).
